# A Case of Inflammatory Pseudotumour Masquerading as Hepatocellular Carcinoma

**DOI:** 10.7759/cureus.45897

**Published:** 2023-09-25

**Authors:** Kalki Rajamanickam Chandrasekaran, Syed Aftab, Issam Al Jajeh, Rajneesh Kumar

**Affiliations:** 1 Gastroenterology and Hepatology, Sengkang General Hospital, Singapore, SGP; 2 Diagnostic Radiology, Sengkang General Hospital, Singapore, SGP; 3 Pathology, Sengkang General Hospital, Singapore, SGP; 4 Gastroenterology and Hepatology, Singapore General Hospital, Singapore, SGP

**Keywords:** igg4 related disease, tumour markers, autoimmune hepatitis, hepatocellular carcinoma, inflammatory pseudotumour

## Abstract

Inflammatory pseudotumors (IPTs) of the liver can mimic malignant lesions. As the name implies, they are usually associated with an inflammatory process and usually regress with the treatment of the underlying pathology. We report a case of a 67-year-old female who presented with right upper quadrant pain, deranged liver enzymes, elevated tumor markers [alpha-fetoprotein (AFP) and CA 19-9], and a large liver mass on imaging, suspected to be hepatocellular carcinoma (HCC). She was eventually diagnosed with IPT complicating the liver inflammation due to autoimmune hepatitis (AIH). She responded well to treatment with steroids and immunosuppressive therapy.

## Introduction

Inflammatory pseudotumors (IPTs) are a rare phenomenon, with an incidence of only about 0.7% [1], that can mimic malignant hepatic neoplasms like hepatocellular carcinoma (HCC) and intrahepatic cholangiocarcinoma [2-4]. The correct identification of the instigating phenomenon is imperative in these cases, as regression of these tumors can occur even after the treatment of the underlying pathology [2-5]. However, the process of diagnosis of the hepatic lesion itself, and the underlying pathology, can be challenging. In this report, we discuss the dilemmas encountered during the diagnostic workup and treatment of one such case.

## Case presentation

**Figure 1:**
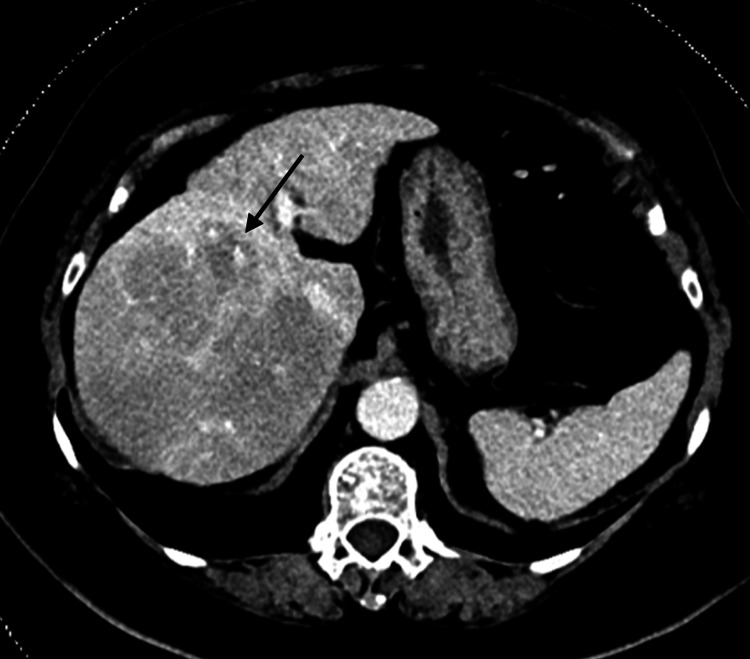
Contrast-enhanced CT abdomen and pelvis (June 2022): PV phase image The PV image shows a heterogeneous mass with margins centered in the right hepatic lobe (black arrow). It demonstrates relatively patchy hypoattenuation when compared with the left lobe of the liver and appears to have some degree of mass-effect CT: computed tomography; PV: portal venous

A 67-year-old female with a past medical history of type 2 diabetes, hypertension, and dyslipidemia was admitted with intermittent right upper quadrant pain, loss of appetite, and 3 kg weight loss over four weeks. She denied alcohol consumption and use of any supplements or traditional medicines and had no family history of liver disease or autoimmune diseases. Her physical examination was unremarkable. The liver enzymes showed a predominantly hepatocellular pattern of liver injury [alanine transaminase (ALT): 207 U/L; alkaline phosphatase (ALP): 123; R factor: 5.1] with normal bilirubin levels. Contrasted CT of the abdomen (Figure 1) revealed heterogeneous enlargement of the entire right hepatic lobe with multiple confluent mass-like hypodensities. Serum alpha-fetoprotein (AFP) level was 83 ug/L and CA 19-9 was 56 U/ml (Table 1).

**Table 1:** Biochemical parameters

Test (serum)	June 2022	August 2022	December 2022	May 2023
	Value (normal range)	Value	Value	Value
Protein	81 g/L (68–85)	70 g/L		72 g/L
Albumin	37 g/L (40–51)	36 g/L		42 g/L
Bilirubin	13 umol/L (7–32)	17 umol/L		13 umol/L
Alkaline phosphatase	123 U/L (39–99)	58 U/L		49 U/L
Alanine transaminase	207 U/L (6–35)	32 U/L		16 U/L
Aspartate transaminase	175 U/L (12–30)	31 U/L		21 U/L
Alpha-fetoprotein	83 ug/L (<7.0)		4.5 ug/L	
CA 19-9	76 U/ml (<34.1)			
Immunoglobulin G	23.15 g/L (5.49–17.11)	12.77 g/L		

**Figure 2:**
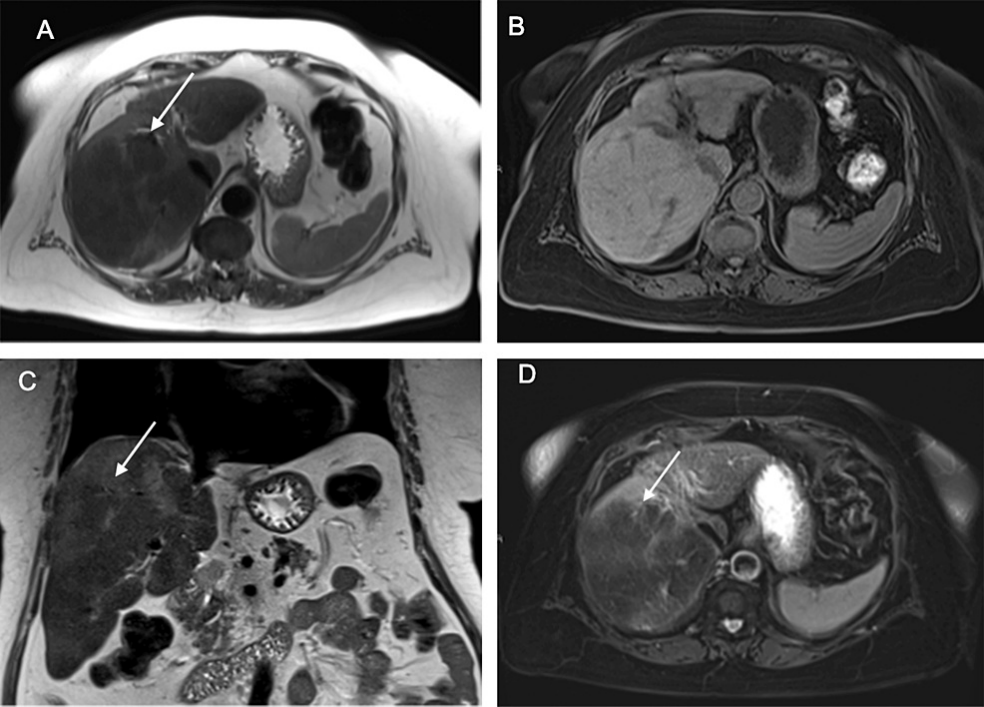
Contrast-enhanced MRI liver (Primovist) (June 2022) The mass appears mildly T2 hypointense relative to the liver parenchyma on the T2-weighted image (A) and T2 fat-saturated image (D). It appears isointense on the T1-weighted image (B). It is difficult to appreciate on the coronal T2-weighted image (C) MRI: magnetic resonance imaging

MRI of the liver (Figure 2) showed a mass-like lobulated appearance of the right lobe, which raised suspicion for either HCC or cholangiocarcinoma, given the red flag symptoms and the elevated tumor markers. The pancreas was normal.

The consensus reached by the multidisciplinary tumor board was to proceed with a liver biopsy to rule out atypical HCC and cholangiocarcinoma, although the imaging features were indeterminate and there was evidence of ongoing liver inflammation. Severe inflammation with possible imaging extinction was considered but disregarded since the right lobe appeared to exert a mass effect on the left lobe.

**Figure 3:**
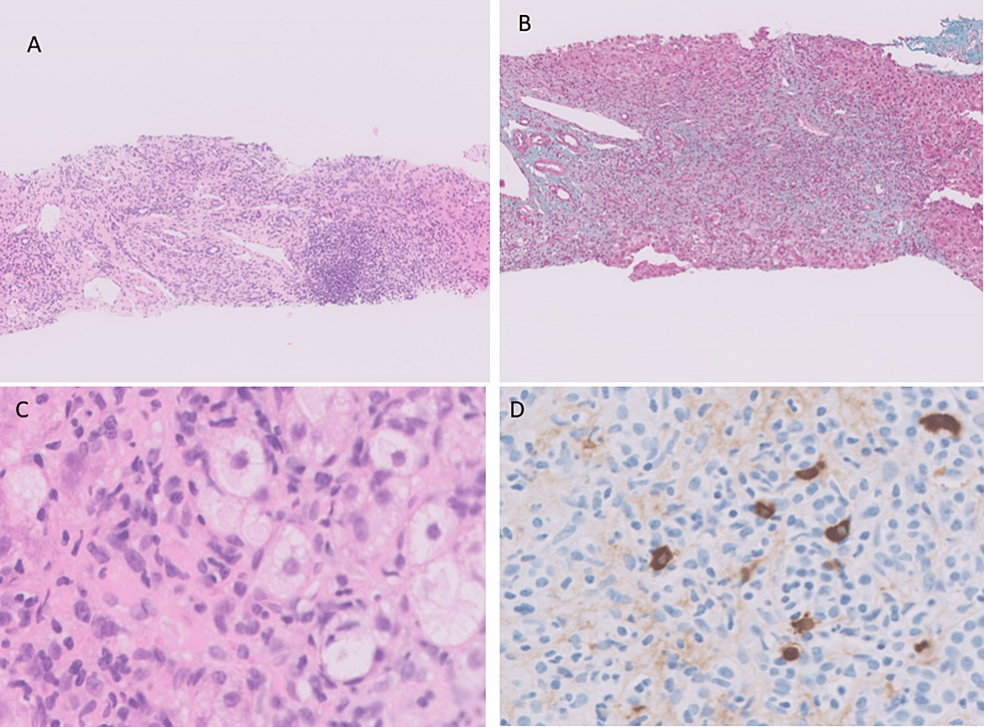
Histology and immunohistochemistry A: Core of liver tissue with portal hepatitis injury pattern (hematoxylin and eosin X2). B: Portal and periportal fibrosis (trichrome stain X2). C: Mild steatosis with rare ballooning (hematoxylin and eosin X20). D: Sparse IgG4 plasma cells (IgG4 immunohistochemistry X20)

Imaging-guided percutaneous liver biopsies were taken from the right lobe and the specimen was deemed adequate, with at least 13 portal tracts visible. Histology (Figures 3A, 3B, 3C) revealed about 10% macrovesicular steatosis with rare ballooning, focal interface inflammation (with mild lymphocytic and plasmacytic infiltration), focal bridging and portal/periportal fibrosis (without evidence of pericellular fibrosis), and absence of bile duct injury or proliferation. Parenchymal necrosis was seen but was focal in distribution. The non-alcoholic fatty liver disease activity score (NAS) was 3. There was no evidence of malignancy. Immunohistochemistry analysis of the tissue showed sparse distribution of IgG4 plasma cells (13 per high power field) for every 100 IgG plasma cells per high power field (Figure 3D). Anti-smooth muscle antibody (ASMA) and antinuclear antibody (ANA) were positive (1:160). The globulin fraction was 40, and the IgG level was elevated at 23. The viral hepatitis screen was negative. The simplified autoimmune hepatitis (AIH) score was 7, and the pre-treatment International Autoimmune Hepatitis Group (IAIHG) score was 18, pointing towards a definitive diagnosis of AIH.

**Figure 4:**
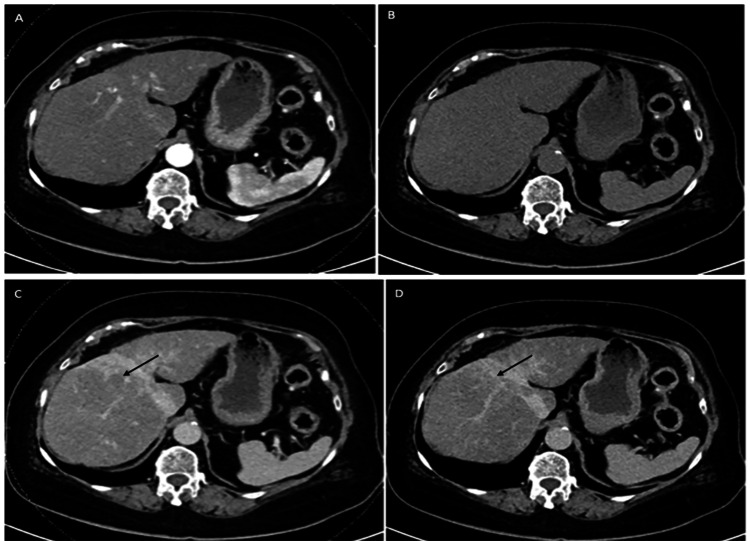
Contrast-enhanced CT liver (November 2022) The mass appears isodense to the liver on the non-contrast image (A). No significant enhancement is appreciated on the arterial phase image (B), nor does it demonstrate appreciable washout on the PV (C) or delayed images (D) CT: computed tomography; PV: portal venous

Having established that the liver mass was neither HCC nor cholangiocarcinoma and given the diagnosis of AIH, the hepatic lesion was diagnosed as an IPT. The elevated AFP was attributed to the ongoing liver inflammation. The patient was started on prednisolone 40 mg once a day, followed by azathioprine 50 mg once a day, with a view to tapering steroids by 5 mg every two weeks, and then maintaining on 5 mg once a day. The liver enzymes and IgG level normalized within three months of initiating treatment (AFP took longer), and the patient continues to fare well now. A quadriphasic CT of the liver (Figure 4) performed about six months later showed that the liver lesion had regressed marginally with treatment. The patient remains under six-monthly ultrasound surveillance to ensure that the lesion regresses completely.

## Discussion

IPT was first described by Brunn in 1939 [6] and it comprises a group of mass-forming lesions that can occur in any organ due to inflammation. Infections (especially of the portal vein), vascular diseases, and autoimmune diseases have commonly been proposed as causative agents [7]. These mass lesions are often mistaken for malignancy [8], especially when confounded by the manner of their presentation and due to the presence of elevated tumor markers, as in our patient. However, elevations in AFP and CA 19-9 are very rare [9,10].

Hepatic IPTs are defined by the WHO classification as benign, non-neoplastic, non-metastasizing masses characterized by the presence of myofibroblastic spindle cells, infiltrated plasma cells, and mixed inflammatory cells without cellular anaplasia or atypical mitoses. The lesions are associated with extensive variability in histologic features, and no uniform criteria have been proposed for diagnosis and classification. In our case, the liver biopsy not only helped to rule out cancer but also to rule in AIH.

In our patient, there are two possibilities for the mass lesion: one involves IPT secondary to AIH, and the other pertains to IPT secondary to IgG4-related liver disease. Interestingly, IgG4-related disease (IgG4-RD) of the liver could present either as biliary strictures or as mass-like lesions [11,12]. The Boston Consensus Criteria for diagnosis of IgG4-RD requires that the patients meet two of the three following major criteria [13]: (a) dense lymphoplasmacytic infiltrate, (b) storiform fibrosis, and (c) obliterative phlebitis. The diagnosis is often supported by immunohistochemistry showing elevated IgG4+ cells and IgG4/IgG ratio greater than 40% [9]. While our patient had lymphoplasmacytic infiltration, she had neither storiform fibrosis nor obliterative phlebitis. Immunohistochemistry analysis for IgG4 plasma cells was unyielding as well. Moreover, unfortunately, we did not have any supporting serum IgG4 levels. Hence, the diagnosis of AIH-related IPT was deemed a better fit as compared to IgG4-related IPT.

The fact that rapid biochemical response was achieved, and has been sustained over the last 12 months, accompanied by the normalization of IgG levels, endorses the diagnosis of AIH-related IPT. We were unable to maintain her on low-dose prednisolone due to issues related to her glycaemic control, which started with the use of steroids, and had to eventually stop it. She remained on a maintenance dose of azathioprine. As the liver inflammation improved, AFP dropped to normal levels and a repeat quadriphasic CT of the liver (the patient declined to undergo a repeat MRI due to claustrophobia) performed six months later showed moderate resolution of the right lobe mass. This contrasts with the course associated with IPTs, which resolve with the resolution of the inflammatory process.

IPTs, in general, have a mean size of about 3-4 cm [13-17], while our patient had a much larger lesion, which was lobulated as well. The modest improvement in the hepatic mass was probably due to its original enormous size, and possibly related to discontinuing steroid therapy earlier than intended. The patient remains under regular imaging surveillance to ensure complete resolution.

## Conclusions

Hepatic IPTs are rare entities, and their diagnosis almost always requires a liver biopsy since imaging features are non-specific. Serum IgG4 level and tissue immunohistochemistry for IgG4 are additional useful tests. More importantly, physicians should not ignore the possibility of other liver diseases that could complicate the scenario and contribute to liver inflammation. It is important to rule out cancer, granulomatous diseases, and infections since their management differs drastically. We believe that our timely diagnosis of AIH and associated IPT saved the patient a lot of psychological trauma and financial loss. Moreover, it is clear that larger IPTs take longer to regress even after the initiation of appropriate therapy. Our case was unique in that two of the tumor markers were elevated, the liver lesion was very large, and regression was taking longer than expected. We hope our case will serve as a source of reference to physicians who encounter such atypical cases in the future.
